# The contribution of reticular basement membrane proteins to basal airway epithelial attachment, spreading and barrier formation: implications for airway remodeling in asthma

**DOI:** 10.3389/fmed.2023.1214130

**Published:** 2023-09-12

**Authors:** Aileen Hsieh, Chen Xi Yang, May Al-Fouadi, Kingsley Okechukwu Nwozor, Emmanuel Twumasi Osei, Tillie-Louise Hackett

**Affiliations:** ^1^Centre for Heart Lung Innovation, St. Paul’s Hospital, Vancouver, BC, Canada; ^2^Department of Anesthesiology, Pharmacology and Therapeutics, University of British Columbia, Vancouver, BC, Canada; ^3^Department of Biology, University of British Columbia, Okanagan, BC, Canada

**Keywords:** basement membrane, extracellular matrix, cell adhesion, lung, epithelial cells, asthma, barrier function

## Abstract

**Rationale:**

In the healthy lung, the pseudostratified conducting airway epithelium is anchored to the reticular basement membrane (RBM) via hemidesmosome junction complexes formed between basal cells and the extracellular matrix (ECM). The RBM within the healthy lung is composed of the ECM proteins laminin and collagen-IV. In patients with asthma, the RBM is remodeled with collagen-I, -III and fibronectin deposition. The goal of this study was to assess the effect of RBM ECM proteins on basal airway epithelial cell attachment, spreading and barrier formation using real-time electrical cell-substrate impedance sensing (ECIS).

**Methods:**

ECIS 8-well arrays were coated with 50 μg/mL of fibronectin, collagen-I, collagen-III, collagen-IV, or laminin and compared to bovine serum albumin (BSA) or uncoated controls. The airway epithelial cell line (1HAEo-) was seeded 40, 50, 60, and 70 k cells/well and continuously monitored over 70 h to assess cell attachment, spreading and barrier formation using high (64 k Hz) and low (500 Hz) frequency resistance and capacitance. Data were analyzed using a one-phase decay model from which half-life (time cells cover half of the electrode area) and rate-constant (cell-spreading rate/h) were determined and a generalized additive mixed effect model (GAMM) was used to assess ECM proteins over the entire experiment.

**Results:**

High-frequency (64 kHz) capacitance measures demonstrated the half-life for 1HAEo-cells to attach was fastest when grown on fibronectin (6.5 h), followed by collagen-I (7.2 h) and collagen-III (8.1 h), compared to collagen-IV (11.3 h), then laminin (13.2 h) compared to BSA (12.4 h) and uncoated (13.9 h) controls. High-frequency (64 kHz) resistance measures demonstrated that the rate of 1HAEo- cell spreading was significantly faster on fibronectin and collagen-I compared to collagen-III, collagen-IV, laminin, BSA and the uncoated control. Low-frequency (500 Hz) resistance measures demonstrated that 1HAEo-cells formed a functional barrier fastest when grown on fibronectin and collagen-I, compared to the other ECM conditions. Lastly, the distance of 1HAEo-cells from the ECM substrates was the smallest when grown on fibronectin reflecting high cell-matrix adhesion.

**Conclusion:**

Airway epithelial cells attach, spread and form a barrier fastest on fibronectin, and collagen-I and these reticular basement membrane ECM proteins may play a protective role in preserving the epithelial barrier during airway remodeling in asthma.

## 1. Introduction

The conducting airway epithelium consists of over 10 molecular and morphologically distinct cell types that have specialized functions along the airway tree that can be classified into three categories: basal, ciliated and secretory cells (1–3). The mucociliary epithelium forms a physical barrier to the inhaled environment through the formation of intercellular adhesion complexes between epithelial cells which include: tight junctions, adhesion junctions, desmosomes, intermediate and gap junctions (4–8). While all pseudostratified airway epithelial cells are in contact with the reticular basement membrane (RBM), it is the basal cells which anchor the airway epithelium to the RBM via hemidesmosomes and enable more superficial cells to attach to the RBM via desmosome complexes (8, 9). Basal cells are ubiquitous within the conducting airways, and there is a direct correlation between the number of basal cells and the thickness of the epithelium (7, 8). Thus, from the large to the small conducting airways, as the epithelial thickness decreases the number of basal cells decreases.

The conducting airway RBM readily seen by light microscopy, was initially shown to be composed of three layers by electron microscopy: the lamina lucida, the lamina densa and the reticular lamina. However follow-up studies demonstrated that the layers were likely due to sample processing and that the reticular basement membrane is actually a single layer (10–13). The key functions of the RBM ECM include providing (1) a structural scaffold to support the adhesion of the epithelium, (2) a template for tissue repair, (3) a reservoir for growth factors, and (4) a physical and permeable barrier for cells and proteins (14). Within the airway RBM, collagen-IV, is the main collagen component and is the major structural component forming a network with laminin, that is stabilized by nidogens, heparin and other proteoglycans (15). When the conducting airway epithelium is damaged by inhaled environmental exposures it can dedifferentiate, flatten and migrate rapidly beneath a plasma-derived provisional matrix containing fibronectin to enable reepithelialization; the underlying mesenchymal cells then deposit collagens-I and –III to provide structure to the ECM during repair and growth factors to stimulate epithelial proliferation and differentiation to restore epithelial barrier integrity (16–20). In the conducting airways of patients with asthma, the RBM is thickened and remodeled with increased deposition of fibronectin and collagen types I and III (15, 21, 22). Further, RBM thickening has been shown to correlate with airway hyper responsiveness and disease severity (23, 24). To date, the functional consequences of alterations in RBM ECM proteins on epithelial functions are incompletely understood.

The goal of the study was to understand the role of different RBM ECM proteins on basal airway epithelial homeostasis in the healthy lung, and provide a reference for studies focused on ECM proteins and epithelial function in lung diseases such as asthma. To understand the effect of RBM proteins (collagen-I, -III, and fibronectin) associated with repair and asthma compared to normal RBM proteins (laminin, collagen-IV) on basal airway epithelial functions, we used Electrical Cell-substrate Impedance Sensing (ECIS), which is a continuous, label free, impedance-based method that enables the study of cell attachment, spreading, and barrier function in the sub-nanometer range (25, 26).

## 2. Materials and methods

### 2.1. Cell culture

The human airway epithelial (1HAEo-) cell line, was formed from airway epithelial cells from a healthy individual which were immortalized using the origin-of-replication defective SV40 plasmid (pSVori-) (27). 1HAEo- cells have been previously shown to maintain tight junctions and desmosomes and polarity by electron microscopy (27), and were used for all experiments at passage 4–7. Cells were grown in Dulbecco’s Modified Eagle Medium, with high glucose (HG-DMEM, GIBCO^®^, 11,965,118, NY, United States) supplemented with 10% Fetal Bovine Serum (CANADA origin, GIBCO^®^, 12,483,020, NY, United States) and 1% Penicillin/Streptomycin/Fungizone solution (HyClone™ 100X Antibiotic Antimycotic Solution, SV3007901, GE Healthcare Life Sciences, United States) in a humidified incubator of 5% CO_2_ at 37°C.

### 2.2. Electric cell substrate impedance sensing

#### 2.2.1. Array preparation

The ECIS Zθ instrument and 8 W10+ arrays (Applied Biophysics Inc., NY, United States), with total of 40 circular 250 μm diameter electrodes situated on inter-digitated fingers in each well, were coated with 50 μg/mL of: Collagen-I (Rat Tail, Natural, 354,236, Corning^®^, NY, United States), Collagen-III (Human, 5,021, Advanced Biomatrix, United States), Collagen-IV (Mouse, Natural, 354,233, Corning^®^, NY, United States), laminin (Mouse, Natural, 354,232, Corning^®^, NY, United States), and fibronectin (Human, Natural, 354,008, Corning^®^, NY, United States) or Bovine serum albumin (BSA, A9418, Sigma, WI, United States). Wells with no coating were used as negative controls and wells with no cells or coating were used for normalization of electrodes and data. After a one-hour incubation at room temperature, arrays were washed with non-supplemented HG-DMEM twice and stabilized with an electrode-stabilizing solution, L-cysteine (200 μL/well, 10 mM L-cysteine in water, applied biophysics, NY, United States) for another hour at room temperature. After washing and adding 500 μL of supplemented media to all wells, the arrays were hooked to the ECIS platform inside the CO_2_ incubator where a second stabilization step that is built into the ECIS software was performed.

#### 2.2.2. Experimental set-up and analysis

After stabilizing the electrodes, cell-free measurements were taken of all wells for 1 h using a multiple frequency mode as recommended by the manufacturer. The run was then paused, and arrays were seeded with 40, 50, 60, or 70 k 1HAEo- cells in each well (except cell-free conditions). The experiment was then run for 72 h and cell resistance and capacitance data were measured in real-time and normalized to cell-free electrode data at time zero (n/n_0_). The cell culture media was changed once after 48 h. To assess cell seeding density, in a parallel set-up, cells were cultured in coated 8-well chambered cell culture slides (MatTek, CCS-8, MA, United States) in a similar manner to ECIS arrays for microscopic observation and cell counting. While we did not test cell death directly in these experiments, we did confirm through visual inspection of the plates that the cell membranes were intact (cobble stone appearance) and there was no cell detachment or rounding.

### 2.3. Statistical analysis

Data from 4 to 7 independent experiments were used for the statistical analysis performed using Graph Pad Prism^®^ 8 (GraphPad Software, Inc. CA, Unites States). One-phase decay and one-phase association exponential models were used to analyze capacitance and resistance data, respectively. *Half-life* (the time required to achieve half-maximal decline/increase in capacitance/resistance of cells growing on different matrices) and *K* (the Spreading rate constant (1/Hr) of these cells) generated from these models were further analyzed for statistical differences using one-way ANOVA followed by Tukey’s multiple comparison test. A *p*-value of ≤0.05 was considered statistically significant. To compare the normalized capacitance and resistance of different coatings over time, a generalized additive mixed model (GAMM) was used which has a random effect component to take into consideration multiple measures per replicate in the longitudinal ECIS data. From the model:


$$\begin{array}{l} Capacitanceorresistance\sim coating+s\left(time\right)+s\left(time:coating\right)\\ +\left(1|Replicate\right) \end{array}$$

we were able to test (1) if there is a coating effect, that is, if the normalized capacitance/resistance (averaged throughout time) is different between each pair of the coatings, (2) if there is a time effect, that is, if the normalized resistance/capacitance changes throughout time, and (3) if there is coating-by-time interaction, that is, if the normalized resistance/capacitance of different coatings change differently between each pair of coatings over time. All the modeling was performed using R package “mgcv” in the R statistical computing environment (version 3.5.0).

## 3. Results

### 3.1. Optimal cell-seeding density for capacitance and resistance measurements

**Figure 1:**
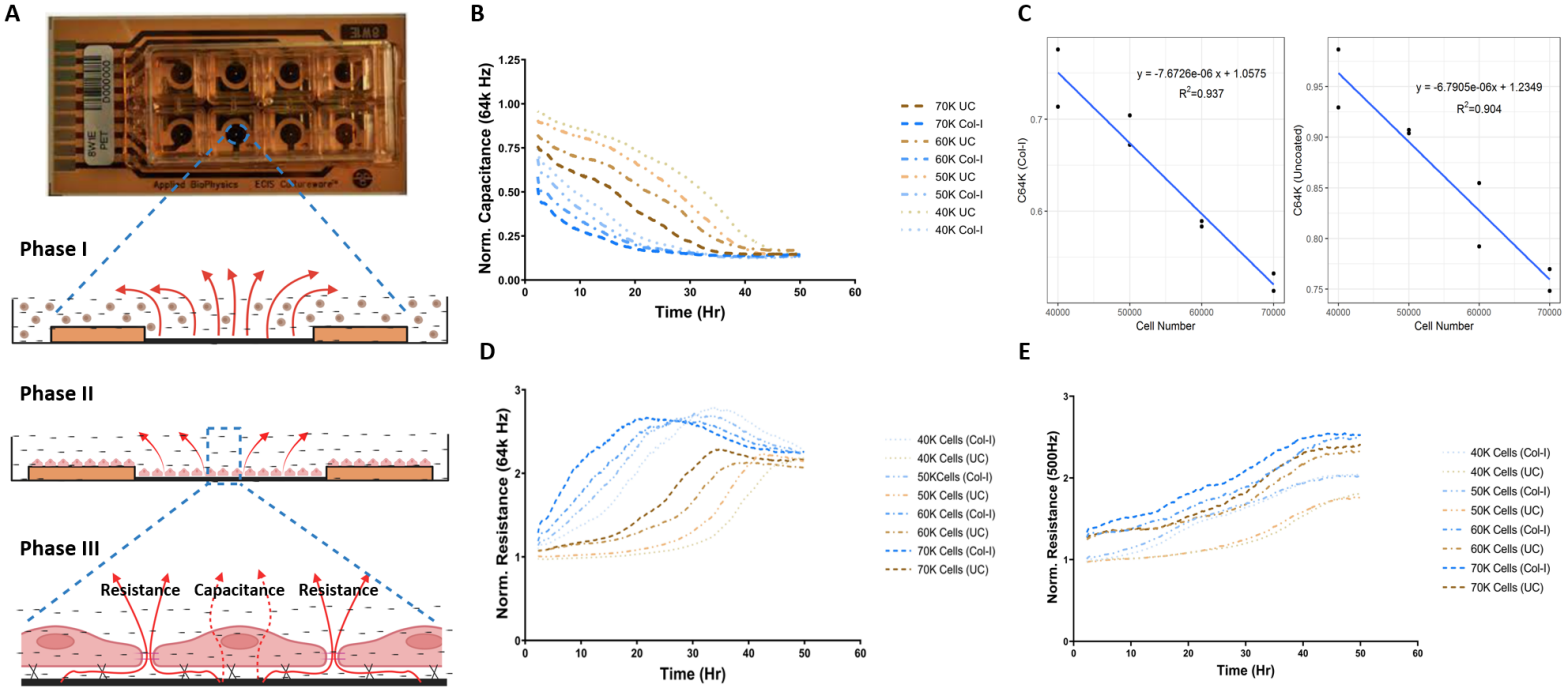
Optimal cell-seeding density for capacitance and resistance measurements. **(A)** Image of ECIS 8 well chamber and illustration demonstrating the phases of epithelial cell attachment, spreading, and barrier function measured through the assessment of capacitance and resistance values (figure created with BioRender.com). 1HAEo- cells were seeded at 40 k, 50 k, 60 k and 70 K cells per well on ECIS electrodes with or without collagen-I and assessed for **(B)** normalized Capacitance values at 64 k Hz to measure cell attachment, **(C)** linear relationship between capacitance and total cell number on collagen-I and uncoated electrodes, **(D)** normalized resistance at 64 K Hz to measure cell spreading, and **(E)** normalized resistance at 500 K Hz to measure barrier function. The data represent 2 well replicates per condition. Values were normalized to the cell-free electrode data at time 0 to account for well-to-well variance.

The human airway epithelial cell line 1HAEo-, was grown in monolayer to model basal airway epithelial cells. To determine the appropriate cell density to measure changes in capacitance (C) and resistance (R) over the time course of the experiment, 1HAEo- cells were seeded 40, 50, 60, 70 k cells per well. Figure 1A illustrates the dynamics of cell adhesion; Phase I cell sedimentation, Phase II cell flattening, Phase III cell spreading and lastly barrier formation in the ECIS® array and electrical current can be used to measure cell–cell and cell-matrix interactions. Transcellular current represented by high-frequency capacitance (64 k Hz), is widely considered a reliable measure of cell attachment (26, 28). Under low resistance frequencies (500 Hz), the majority of the current passes under and in between cells (paracellular current), and thus represents a good measure of barrier function, whereas high resistance frequencies (64 k Hz) are a good measure of cell spreading (26, 28).

As shown by the normalized capacitance values (64 k Hz) in Figure 1B, 1HAEo- cell attachment was fastest on collagen-I coated electrodes for all cell seeding densities compared to uncoated electrodes (*p* < 0.05) and 1HAEo- cell coverage was fastest in wells seeded with 70 k cells and lowest in wells with 40 k cells. The validity of the ECIS data was confirmed by a linear correlation between capacitance (64 k Hz) values and the physical cell counts of 1HAEo- cells in collagen-I and uncoated conditions (Figures 1B,C, *R*^2^ = 0.94 and 0.90 respectively). Figure 1D shows the normalized resistance values for 64 kHz, which demonstrates 1HAEo- cell spreading was greater with higher seeding densities (60 and 70 k cells) compared to the lower seeding densities (40 and 50 k cells) on collagen-I coated compared to uncoated electrodes. Lastly, the normalized resistance values for 500 Hz showed that cell–cell contact representing the formation of barrier function was greater with higher seeding densities (60 and 70 k cells) compared to lower seeding densities (40 and 50 k cells) on collagen-I coated compared to uncoated electrodes (Figure 1E). Hence for all other experiments, we choose a 1HAEo- cell seeding density of 70,000 cells.

### 3.2. Airway epithelial cell attachment on different RBM extracellular matrix proteins

**Figure 2:**
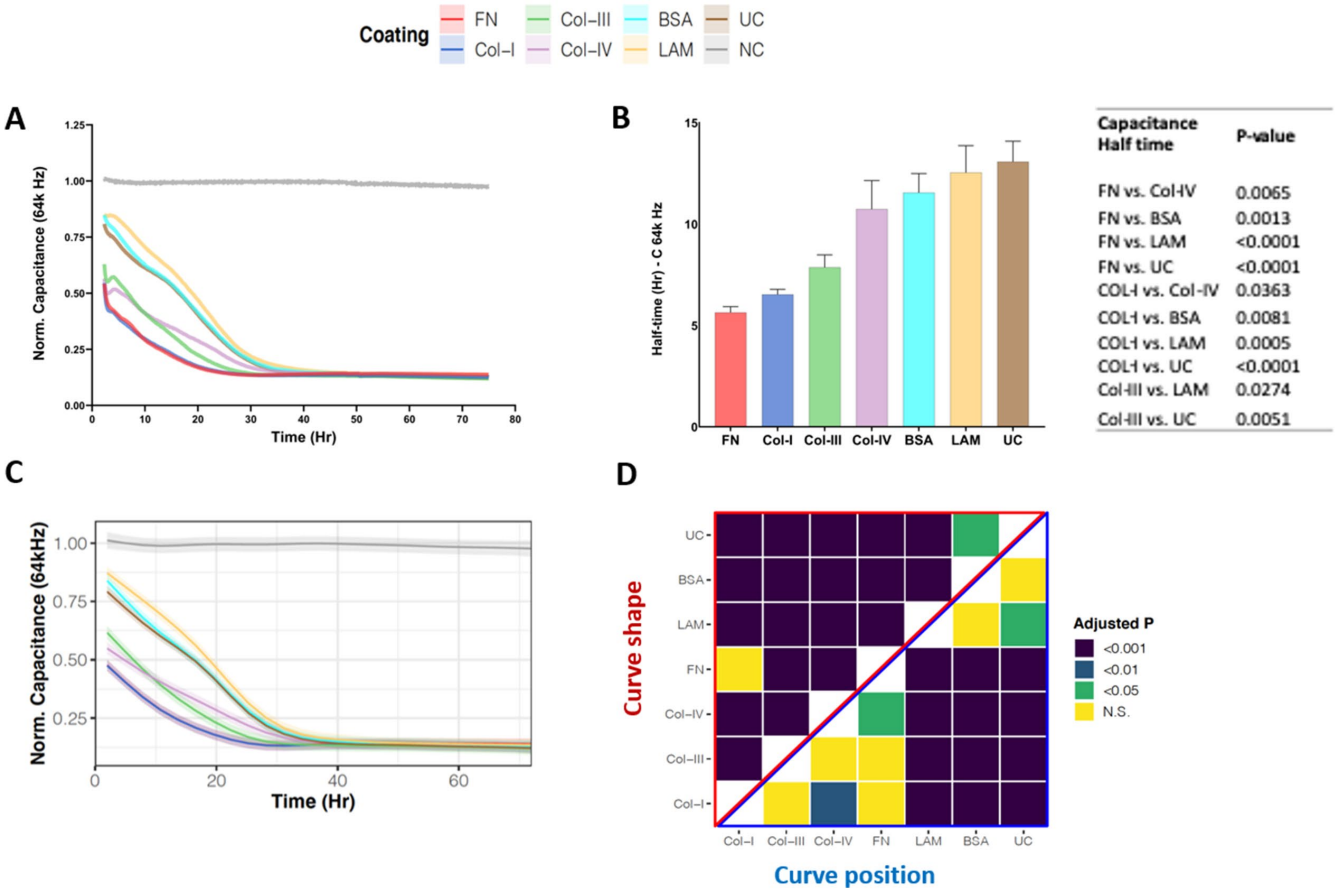
Airway epithelial cell attachment on different reticular basement membrane extracellular matrix proteins. 1HAEo- cells were seeded at 70 K cells per well on ECIS electrodes coated with 50 μL/mL of fibronectin (FN, red), collagen-I (Col-I, Blue), collagen-III (Col-III, green), collagen-IV (Col-IV, purple), bovine serum albumin (BSA, turquoise), or laminin (LAM, yellow) and compared to uncoated electrodes (UC, brown) or electrodes with no-cells (NC, grey). 1HAEo- cells were measured in real-time for 70 h and assessed for **(A)** normalized capacitance (64 k Hz) to assess cell attachment, **(B)** for each ECM protein condition the half-life (Hr, hours) of capacitance (64 k Hz) was calculated using a one-phase association model (values shown are mean ± SEM). A one-way ANOVA with Tukey’s *post-hoc* test was used and the table shows all significant *p*-values. **(C)** The curves represent the GAMM-modeled normalized capacitance (64 K Hz) for each hour of the experiment and the shading denotes the 95% confidence interval. **(D)** The heatmap shows the adjusted-*p* value (Benjamini-Hochberg False Discovery Rate) from the GAMM-model. The upper part (red triangle) compares how the capacitance of each pair of ECM proteins changes differently over time (curve shape) and the lower part (blue triangle) compares the average capacitance of each pair of ECM proteins (curve position). The data represent *n* = 4–7 experiments, with 2 well replicates per condition. Values were normalized to the cell-free electrode data at time 0 to account for well-to-well variance.

We utilized high frequency capacitance (64 k Hz), to quantify the behavior of basal epithelial cell attachment when exposed to different RBM ECM proteins. Figure 2A shows the normalized capacitance (64 k Hz) curves for 1HAEo- cells grown on normal RBM proteins collagen-IV and laminin, versus asthma-associated RBM proteins fibronectin, collagen-I and -III, a non-specific binding control bovine serum albumin (BSA) and uncoated electrodes. As shown in Figure 2B, the half-life to cover the electrodes was shortest when 1HAEo- cells were grown on fibronectin, collagen-I and-III compared to collagen-IV, laminin, BSA and uncoated controls. To enable assessment of cell attachment over the entire time course of the experiment we used a generalized additive mixed effect model to directly compare the capacitance (64 k Hz) curve shape and position for each ECM protein. The line graph in Figure 2C shows the GAMM-modeled capacitance (64 k Hz) for each hour of the experiment. The lower the position and the steeper the slope of the curve indicates that the capacitance (64 Hz) dropped the fastest when 1HAEo- cells grew on an ECM protein. The heatmap in Figure 2D shows the adjusted-*p* value [Benjamini-Hochberg False Discovery Rate (BH-FDR)] from the GAMM model. The upper part (red triangle) of the heatmap compares how the capacitance of each pair of ECM proteins changes differently over time and the lower part (blue triangle) compares the average capacitance of each pair of ECM proteins. In this analysis, 1HAEo- cells grown on fibronectin and collagen-I had the fastest coverage, then collagen-III and-IV, and on uncoated, BSA or laminin 1HAEo- cells had the slowest coverage. These data demonstrate that 1HAEo- cells have a greater preference for fibronectin and fibrillar collagen-I, for cell attachment.

### 3.3. Airway epithelial cell spreading on different RBM extracellular matrix proteins

**Figure 3:**
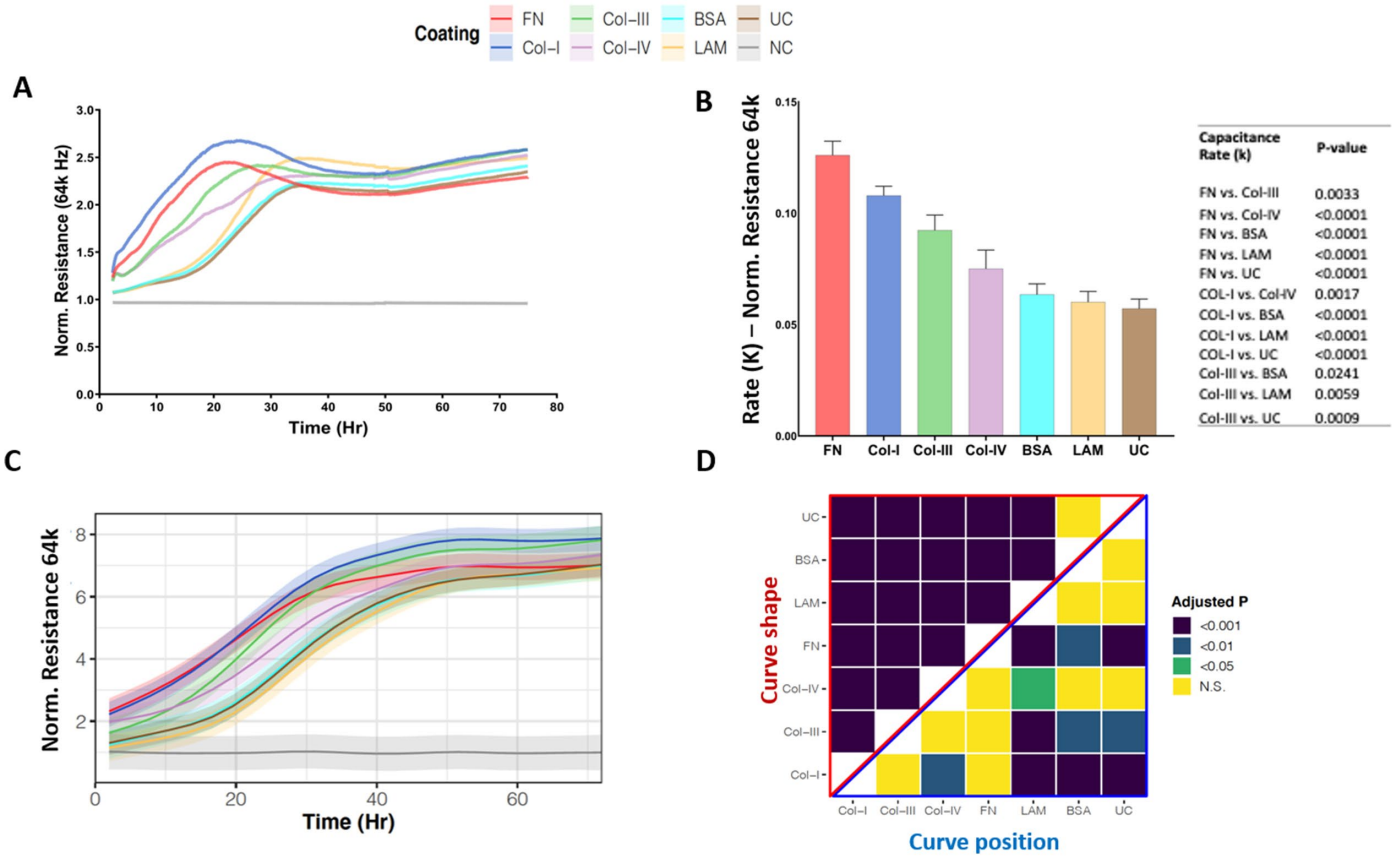
Airway epithelial cell spreading on different reticular basement membrane extracellular matrix proteins. 1HAEo- cells were seeded at 70 K cells per well on ECIS electrodes coated with 50 μL/mL of fibronectin (FN, red), collagen-I (Col-I, Blue), collagen-III (Col-III, green), collagen-IV (Col-IV, purple), bovine serum albumin (BSA, turquoise), or laminin (LAM, yellow) and compared to uncoated electrodes (UC, brown) or electrodes with no-cells (NC, gray). 1HAEo- cells were measured in real-time for 70 h and assessed for **(A)** normalized resistance (64 k Hz) to assess cell spreading, **(B)** the rate constant (K, (1/Hr)) for spreading of 1HAEo- cells grown on different matrices were generated by applying a one-phase decay exponential model of these cells (values shown are mean ± SEM). A one-way ANOVA and Tukey’s *post-hoc* test was used and the table shows all significant p-values. **(C)** The curves represent the GAMM-modeled normalized resistance (64 k Hz) for each hour of the experiment using a generalized additive mixed model and the shading denotes the 95% confidence interval. **(D)** The heatmap shows the adjusted-p value (Benjamini-Hochberg False Discovery Rate) from the GAMM-model. The upper part (red triangle) compares how the resistance of each pair of ECM proteins changes differently over time (curve shape) and the lower part (blue triangle) compares the average resistance of each pair of ECM proteins (curve position). The data represent *n* = 4–7 experiments, with 2 well replicates per condition. Values were normalized to the cell free electrode data at time 0 to account for well-to-well variance.

Figure 3A, shows the normalized high-frequency resistance (64 k Hz) values for 1HAEo- cells seeded on the different RBM ECM proteins which allow for the assessment of cell spreading. Figure 3B shows the rate constant (k), which is the rate (1/Hr) of cell spreading over the different ECM proteins assessed. 1HAEo- cells spread significantly faster on fibronectin compared to collagen-III, collagen-IV, BSA, laminin and uncoated controls. In addition, cells growing on collagen-I spread significantly faster than collagen-IV, BSA, laminin and the uncoated control. Lastly collagen-III spread significantly faster than laminin and the uncoated control. To assess cell spreading over the entire experiment, the line graph in Figure 3C shows the GAMM-modeled high-frequency resistance (64 k Hz) for each hour of the experiment. The lower the position and the steeper the slope of the line indicates that the resistance (64 k Hz) increased the fastest when 1HAEo- cells spread on an ECM protein. The adjusted-*p* value heatmap in Figure 3D, confirmed that 1HAEo- cells spread the fastest on fibronectin and collagen-I over the time course of the experiment compared to collagen-IV, laminin, BSA and the uncoated control.

### 3.4. Airway epithelial barrier formation on different RBM extracellular matrix proteins

**Figure 4:**
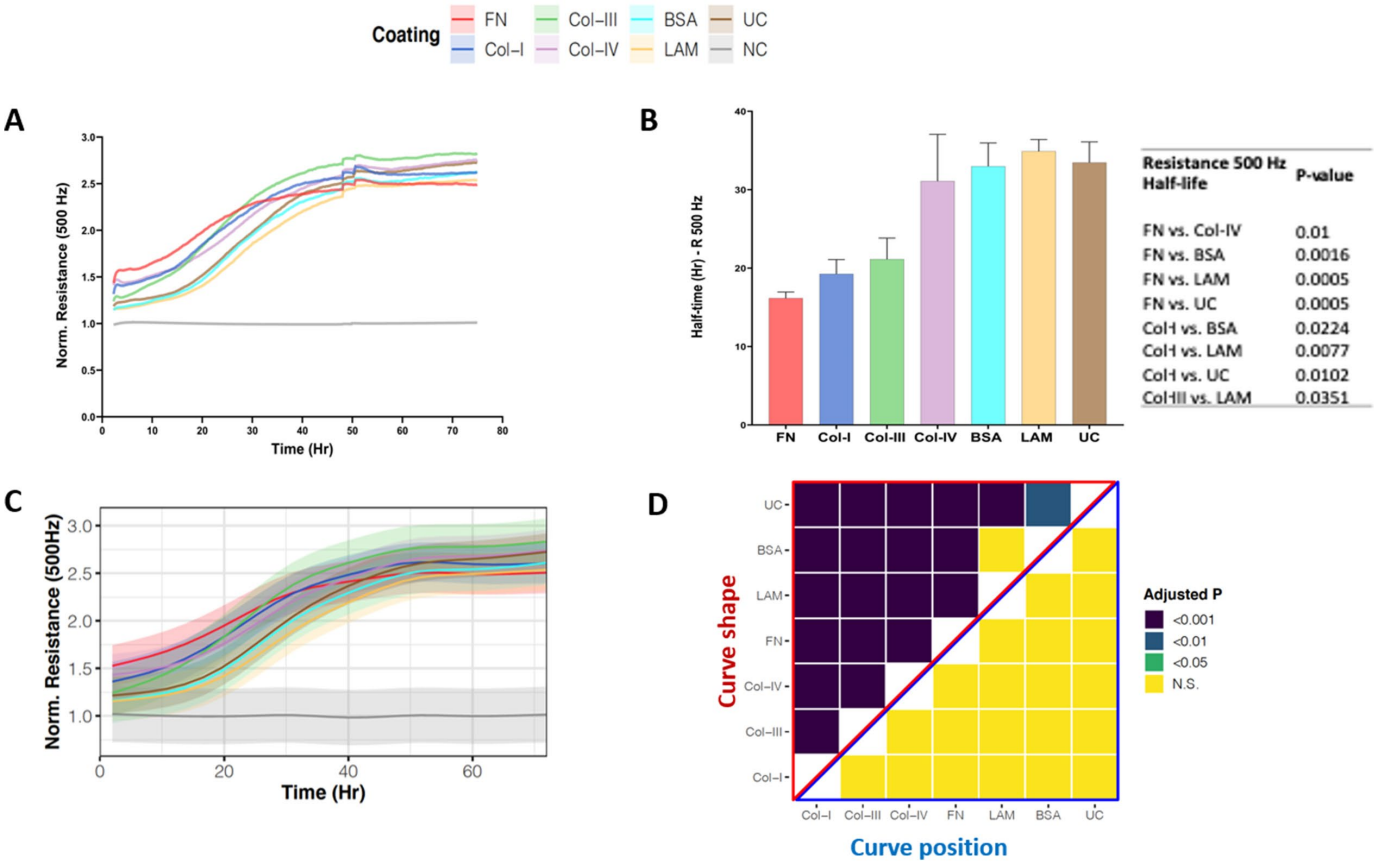
Airway epithelial barrier formation on different reticular basement membrane extracellular matrix proteins. 1HAEo- cells were seeded at 70 K cells per well on ECIS electrodes coated with 50 μL/mL of fibronectin (FN, red), collagen-I (Col-I, Blue), collagen-III (Col-III, green), collagen-IV (Col-IV, purple), bovine serum albumin (BSA, turquoise), or laminin (LAM, yellow) and compared to uncoated electrodes (UC, brown) or electrodes with no-cells (NC, gray). 1HAEo- cells were measured in real-time for 70 h and assessed for **(A)** normalized resistance (500 Hz) to assess cell spreading, **(B)** for each ECM protein condition the half-life (Hr, hours) to form a complete barrier measured by resistance (500 Hz) was calculated using a one-phase association model (values shown are mean ± SEM). A one-way ANOVA with Tukey’s *post-hoc* test was used and the table shows all significant *p*-values. **(C)** The curves represent the average normalized resistance (500 Hz) for each hour of the experiment using a generalized additive mixed model and the shading denotes the 95% confidence interval. **(D)** The heatmap shows the adjusted-p value (Benjamini-Hochberg False Discovery Rate) from the GAMM-model. The upper part (red triangle) compares how the resistance of each pair of ECM proteins changes differently over time (curve shape) and the lower part (blue triangle) compares the average resistance of each pair of ECM proteins (curve position). The data represent *n* = 4–7 experiments, with 2 well replicates per condition. Values were normalized to the cell-free electrode data at time 0 to account for well-to-well variance.

Figure 4A, shows the normalized low-frequency resistance (500 Hz) values for 1HAEo- cells seeded on the different RBM ECM proteins which allow for the assessment of cell junction formation as a measure of barrier function. Within 50 h, 1HAEo- cells seeded on all RBM ECM proteins reached the same resistance, indicating that cells were fully-confluent and had formed a functional barrier. When we assessed the half-life of cells to form a functional barrier, fibronectin and collagen-I had the shortest half-life to form a functional barrier compared to collagen-IV, laminin, BSA and uncoated controls and collagen-III had a faster half-life than laminin (Figure 4B). The line graph in Figure 4C shows the GAMM-modeled low-frequency resistance (500 Hz) for each hour of the experiment. The adjusted-*p* value heatmap in Figure 4D, confirmed that the rate (curve shape) at which 1HAEo- cells form a barrier is the fastest for fibronectin, collagen-I and –III compared to collagen-IV, laminin, BSA and the uncoated control, but there is no difference in the epithelial barrier formed (curve position) over the time course of the experiment (Figure 4D, 0.05 FDR).

### 3.5. The physical interaction of cells with different RBM extracellular matrix proteins

**Figure 5:**
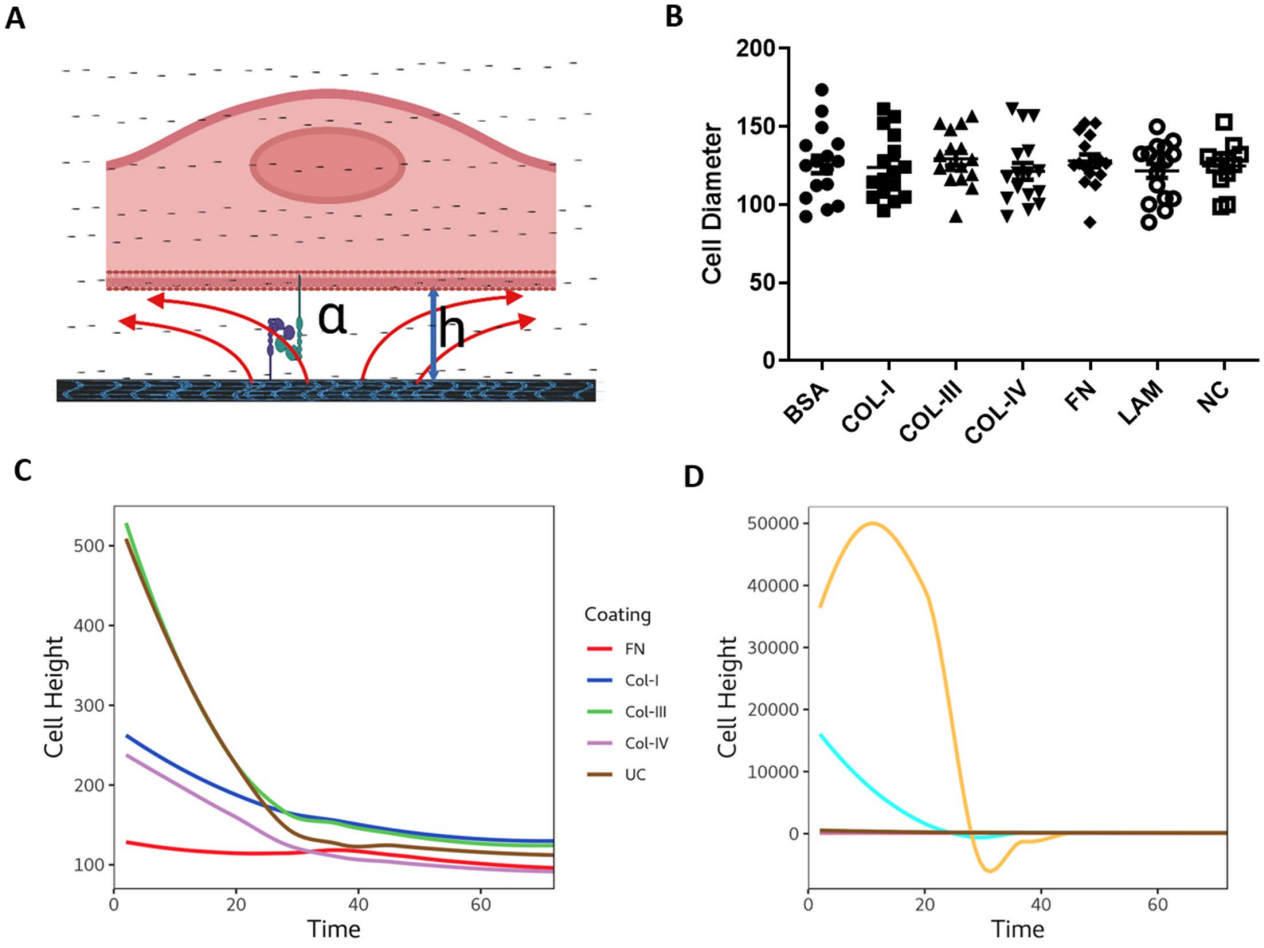
The physical interaction of cells with different reticular basement membrane extracellular matrix proteins. **(A)** Illustration demonstrating epithelial cell attachment and distance from the extracellular matrix protein (figure created with BioRender.com). 1HAEo- cells were seeded at 70 K cells per well on ECIS electrodes coated with 50 μL/mL of fibronectin (FN, red), collagen-I (Col-I, Blue), collagen-III (Col-III, green), Collagen-IV (Col-IV, purple), bovine serum albumin (BSA, turquoise), or Laminin (LAM, yellow) and compared to uncoated electrodes (UC, brown) or electrodes with no-cells (NC, gray) and measured for **(B)** Cell diameter using light microscopy (mean ± SD), **(C)** Cell height calculated from the cell radius and alpha values using: h = ρ/(α^2^ × r^2^) Cell height at 72 h. Repeated measure ANOVA was used for statistical analysis.

To understand the interaction of 1HAEo- cells with the RBM ECM components we calculated the distance between cells and the underlying matrix by calculating the height (h) or distance between cells and electrode surface using the following equation: h = ρ/(α^2^ × r^2^), where ρ is the resistivity of the medium, α (cm.ohm0.5) reflects the cell-matrix adhesion, and Rb (ohm.cm2) reflects the cell–cell interactions as previously described (29–32). Figure 5A illustrates cell-matrix adhesion with the distance between the cell and the electrode labeled as “h” (height). As shown in Figure 5B the cell diameter of 1HAEo-s measured using light microscopy was not different when seeded and grown on the difference ECM conditions. As shown in Figure 5C, the distance between the electrode and the cell was smallest for fibronectin, then collagen-1 and -IV, compared to collagen-III and the uncoated control whereas, BSA and laminin coated electrodes had the greatest distance to the cell membrane (Figure 5D).

## 4. Discussion

In this study, we report that human basal airway epithelial cells adhere, migrate and form a functional barrier more rapidly on the ECM substrates fibronectin and fibrillar collagen-I associated with RBM repair compared to collagen-IV and laminin which are normally found within the healthy RBM. These data form the first comprehensive review of the effect of different basement membrane ECM substrates on airway epithelial attachment, spreading and barrier function using real-time, electrical cell-substrate impedance sensing within the sub-nanometer range. These data have important implications for our understanding of the RBM ECM in epithelial wound repair and how remodeling of the RBM may influence epithelial functions in many respiratory diseases such as asthma.

The most fundamental property of epithelial cells is the generation of an apical domain, which interacts with the external environment and the basolateral domain to enable contact with the reticular basement membrane and neighboring cells. With millions of individual epithelial cells within an epithelium it is essential that each cell makes the right decision when to divide, migrate, differentiate, or die. Tight control of these cellular decisions is guided by the mechanical cues from the RBM during epithelial homeostasis and repair. We report for the first time using ECIS that airway epithelial cells attach more rapidly to fibronectin, and fibrillar collagen-1, which are associated with repair and remodeling of the RBM in asthma compared to collagen-IV and laminin which normally form the RBM (15, 21, 22). As cell attachment is a prerequisite for cellular survival and growth (33), the effect of RBM collagen-IV and laminin on cell attachment has been studied since the early 1980s. These studies using radioimmunoassays to assess cell attachment demonstrated that in various cell lines that cell adhesion is promoted primarily by collagen-IV compared to laminin (34, 35). Indeed, our data support these findings demonstrating increased attachment with collagen-IV compared to laminin. Regarding the effect of ECM substrates associated with repair, Hamilton and colleagues, recently investigated the effect of ECM substrates on airway epithelial attachment and differentiation in bioengineered grafts. As part of their study, they investigated the attachment of primary epithelial cells to tissue culture plates coated with collagen-I, collagen-IV, fibronectin, vitronectin and laminin within 30 min using fluorescence cell counting (36). While the authors reported that collagen-IV had greater attachment than laminin in their assay, fibronectin and collagen-I also demonstrated a similar enhanced attachment compared to laminin at 30 min (36). Our data assessed cell attachment using ECIS over 72 h and show the half-life of 1HAEo- cells to cover the electrodes for fibronectin, collagen-I and –III was 6.5, 7.2, 8.1 h respectively, compared to collagen-IV (11.3 h) and laminin (13.2 h) which were within the same range of BSA coated (12.4 h) and uncoated (13.9 h) controls. Our study highlights the importance of being able to study the cells in real-time in order to assess the dynamics of cell attachment which was completed in all ECM conditions at 30 h. This is important, as the process of cell adhesion is characterized by three stages which include; phase I initial attachment of the rounded cell by sedimentation, phase II flattening and attachment of the cell, and Phase III cell spreading and stable adhesion through structural reorganization of the cell cytoskeleton (37, 38) which takes longer than 30 min (Figure 1A). In further support of our findings, several studies have previously reported that fibronectin and collagen-I enhance smooth muscle cell proliferation whereas cells grown on laminin divide more slowly, which may indicate why there is more smooth muscle mass in the airways of asthmatic patients (39, 40). The authors proposed the mechanism behind this response was through the upregulation of the nuclear proliferation marker Ki67 expression. While we did not assess Ki67 expression directly in our study, we did confirm that the measurement of capacitance (64 k Hz) correlates highly with cell number and that the capacitance (64 k Hz) increased the greatest when cells were seeded on fibronectin and collagen-I, indicating more cells present. Previous studies of the airway epithelium of asthmatic patients have demonstrated increased numbers of CK5+/p63+ basal cells, and SP progenitor cells and increased expression of Ki67^23,64^ (41). While no mechanism for increased numbers of basal cells with Ki67 expression has been proposed in asthma, in support of this hypothesis, fibronectin has been shown to induce cell proliferation and inhibit apoptosis in the human bronchial epithelial cell lines BEAS-2B and 16-HBEs (42). Thus, future work to assess the effect of elevated fibronectin and collagen-I in the RBM of asthmatic patients on Ki67 expression in primary airway epithelial cells would determine if this association is causal or not.

With regards to airway epithelial cell spreading, we found fibronectin and fibrillar collagen-1 demonstrated an enhanced rate of 1HAEo- cell spreading compared to collagen-IV, laminin and BSA controls. In support of our findings, Mereness et al. previously reported using light microscopy that normal human bronchial epithelial cells and primary human pediatric lung epithelial cells spread more over a 3 h timepoint, when grown on either collagen-I and-VI (Collagen-6) versus Matrigel, for which the primary components are: laminin (~60%), collagen-IV (~30%), entactin (~8%) and the heparin sulfate proteoglycan perlecan (~2–3%) (43). Further, Garat and colleagues, using video microscopy experiments, demonstrated that following a scratch wound, alveolar epithelial cells spread and migrate the fastest when grown on fibronectin compared to collagen-I and collagen-IV (44). Compared to the other reported studies ECIS provides the opportunity to assess the cells during the entire experiment so that the rate of cell spreading could be assessed. In terms of the mechanisms for cell attachment and spreading, we did not assess in this study the alterations in integrin expression or localization which are known to be important in hemidesmosome formation with the cell cytoskeleton, due to limitations of imaging the ECIS arrays during the time course of the experiment. In our study, we did assess the effect of the ECM substrates on cell size and height from the electrode and found cells coated on fibronectin had the least distance from the electrode, indicating an earlier and tighter attachment representing Phase III of cell attachment with flattening and stable adhesion through structural reorganization of the cell cytoskeleton. In support of our findings, Kligys et al., have previously shown with real-time fluorescent imaging that fibronectin impedes the migration of normal bronchial epithelial cells grown on a mixture of laminin and fibronectin and thus enhances their “stickiness” (45).

Lastly, we assessed epithelial barrier function, and found fibronectin and collagen-I significantly decreased the half-life to form a confluent epithelial barrier when measured by low frequency resistance (500 Hz), but all ECM substrates were able to form a patent barrier by 45 h. While no study has directly compared the effect of ECM substrates on epithelial barrier function using ECIS, previous studies using the SV40-transformed bronchial epithelial cell line, 16HBEs, demonstrated a similar finding that on uncoated electrodes it took 70 h to form a confluent monolayer when measured with low frequency resistance (400 Hz) when seeded with 75 K cells (26). Using single, transepithelial resistance measurements using an ohmmeter, Koval and colleagues more recently showed that rat alveolar epithelial cells grown on fibronectin compared to collagen-I and laminin had greater trans-epithelial resistance measures, however after 5 days of culture, alveolar epithelial cells grown on laminin had the greatest transepithelial resistance (46). Together these studies highlight the importance of being able to measure the functional capacity of cells over a significant time course of days or weeks to understand how cells respond to their ECM environment.

In diseases such as asthma, abnormal thickening and remodeling of the RBM develops early in the disease course and is observed in children and adults with mild to severe and fatal asthma, and persists after remission (47, 48). It has been shown that thickening of the airway RBM involves the accumulation of collagens I, III and fibronectin (49). While it has been previously proposed that thickening of the airway RBM may lead to defective cross-talk in the epithelial-mesenchymal trophic unit leading to increased inflammation and fibrosis, the data reported in this study highlight that increased deposition of fibronectin and fibrillar collagen-I could potentially be beneficial and enhance attachment of the damaged and defective airway epithelium observed in patients with asthma (41, 50, 51). These data demonstrate that future studies using ECIS will be helpful to determine if there are alterations in airway epithelial attachment, spreading and barrier function, and how this may be altered by the RBM ECM in diseases such as asthma.

While the use of ECIS enabled assessment of cell adhesion, spreading and barrier function in real-time within the same experiment, there are some limitations to note for the study. Firstly, the ECM substrates were studied individually, whereas within the RBM ECM proteins would exist as a matrix with a 3-dimensional organization. While it is not possible to conduct sub-nanometer range ECIS measurement in 3D cultures, further monolayer studies would be beneficially to determine if the combination of ECM proteins may have a greater influence on epithelial cell function. Secondly, in this study we used soluble human-plasma derived fibronectin. Fibronectin is 500 kDa dimeric glycoprotein with multiple plasma soluble and cellular derived isoforms including three splice variants (52). Cellular fibronectin, has been shown to be 50 times more potent than soluble fibronectin at inducing cell migration and wound repair (53), and is most prominently produced by fibroblasts, chondrocytes, and smooth muscle upon injury to modulate re-epithelization and wound repair (52). Further studies comparing soluble and cellular fibronectin will be helpful to determine the most effective ECM substrates to support epithelial repair and regeneration in grafts. Lastly, this study utilized the human SV40 transformed 1HAEo- epithelial cell line known to maintain epithelial barrier function characteristics, and while the results are comparable to other studies using normal human bronchial epithelial cells it will be important in future studies to determine if there are differences in epithelial cells derived from patients with respiratory diseases such as asthma.

In summary, electrical cell-substrate impedance sensing provides real-time and sensitive measurement to study airway epithelial cell attachment, spreading and barrier formation on different ECM proteins. We found that the RBM ECM substrates fibronectin and collagen-I, which are present within the repairing RBM cause the fastest rate of airway epithelial cell attachment, spreading and barrier formation. As the RBM in individuals with asthma contains increased deposition of fibronectin and collagen-I, we propose that this remodeling may be a protective mechanism to maintain the epithelial integrity of the damaged airway epithelium in asthmatic patients. In future studies, electrical cell-substrate impedance sensing provides an opportunity to assess cell interactions with their ECM microenvironment to study tissue remodeling in the setting of different respiratory diseases with alterations in ECM such as asthma, chronic obstructive disease and idiopathic pulmonary fibrosis.

## Data availability statement

The raw data supporting the conclusions of this article will be made available by the authors, without undue reservation.

## Author contributions

MA-F, EO, and T-LH conceptualized and designed the experiment. MA-F and AH performed the experiments. MA-F, CY, AH, and T-LH analyzed and interpreted the results. MA-F, CY, KN, and T-LH prepared the figures. MA-F, EO, KN, and T-LH drafted the manuscript. T-LH reviewed the manuscript. All authors read and approved the final version.

## Conflict of interest

The authors declare that the research was conducted in the absence of any commercial or financial relationships that could be construed as a potential conflict of interest.

## Publisher’s note

All claims expressed in this article are solely those of the authors and do not necessarily represent those of their affiliated organizations, or those of the publisher, the editors and the reviewers. Any product that may be evaluated in this article, or claim that may be made by its manufacturer, is not guaranteed or endorsed by the publisher.
